# How I Was Relieved of Locomotor Ataxia

**Published:** 1905-12

**Authors:** Clem C. Greene

**Affiliations:** Atlanta, Ga.


### HOW I WAS RELIEVED OF LOCOMOTOR ATAXIA.

BY CLEM. C. GREENE, M.D.
ATLANTA, GA.

It is not the purpose of this paper to discuss the cause or causes of locomotor ataxia, but to deal with the affection from the point of view expressed in the foregoing caption. That I was treated by the usual methods and without results, some of the best-known physicians here and throughout the country can affirm.

From May, 1897, to the early part of 1903, I was the victim of locomotor ataxia, and from first to last was under the care of some of the best-known physicians in the country. Without exception the alternating treatment of mercury and the iodides was recommended and was faithfully carried out. The results were, however, uniformly unsatisfactory. There was neither amelioration of symptoms nor arrest of the disease.

After the usual therapeutic methods had been exhausted, early in the year 1903 I had recourse to the Ultra Violet Light treatment. At this time I moved about with difficulty, suffering from edema of the feet, limbs and body, rapid respiration, and all the other distressing, annoying and painful symptoms of what was apparently the last stage of tabes dorsalis.

Before entering into the details of light therapy, it would be well to consider the effect of light upon the human body. The following explanation is offered in lieu of a better, with the frank acknowledgment that it may or may not be the correct one:

We are taught that in nature there is nothing still. Light, itself a mode of motion, intensifies the molecular motion in proportion to the rapidity and length of light waves—the more intense the rays, the greater their effect. Again, it has been shown that of all the colors of the solar spectrum the violet is the only germicidal or life-destroying color, and the red are the heat or life-giving rays. This has been shown in the reaction of both plant and animal life; under both of these rays the duration of life is briefer under the influence of the rays from the violet end of the spectrum, longer under those from the red end. The effect of the former class is germicidal and energizing to normal cell life.

MODE OF CONDUCTING THE TREATMENT.

The apparatus employed is a cabinet about seventy-two inches long, fifty high and thirty-six broad, lined with mirrors and having at each end a 110 volt arc light. Between these lights, and as near as is comfortable, the patient sits. The carbons used in the arc lamp are cored and filled with such elements as yield the greatest number of actinic rays. It is not possible, as yet, to secure carbons giving an absolutely blue light, but the manufacturers have succeeded in offering a material that appears to fulfill the requirements, shows a minimal quantity of rays of lesser defrangibility. With such carbons I have been enabled to secure some very interesting and striking results.

The patient divested of all clothing sits between the lights, with his head above the top of the cabinet. As heat is not the quality desired the top of the cabinet is in part removed and the space covered with thin white cloth, which is also carried around and under the patient's face and neck. The patient soon responds to the effect of the light in a generous perspiration. The perspiration is not due to the temperature of the air in the cabinet, as the latter is perfectly ventilated and the heat generated by the lamps readily passes off through the thin cloth over the opening—the temperature within the cabinet at no time exceeds 103° F. Compare this with the temperature of 150° to 170° of the Turkish bath. In such establishments the bather is expected to exist for an hour or more in such a temperature, breathing stale air which has probably already done duty for 50 or 100 other unfortunates. In the light bath the patient sits quietly and alone for fifteen to twenty-five minutes, his body in an atmosphere of 101, 102, or 103 degrees and breathing clean fresh air. The secretion from the sweat-glands is aroused by the direct rays of light acting somewhat in the manner of the X-rays, in that they know no resistance and pass through soft and osseous tissues alike. The light creates or intensifies molecular activity chemically, through the medium of the violet rays. The violet light being germicidal hastens the effect by causing a more rapid dissolution or disassociation of the diseased elements from the normal tissue. We must remember that two conditions of the human body are alone possible—the healthy and the unhealthy. The diseased cells are less resistent and undergo involution under the vibratory movement of molecular action as occasioned by the passage of the violet and ultra violet rays. They are converted into a serous or fluid condition, and nature, ever careful of the surplus or the effete, disposes of them by absorption or elimination through the emunctories, including the skin.

After a free perspiration the patient is taken from the cabinet, dried, rubbed, massaged and then exposed to the light from an additional arc lamp. The lamp is similar to the type used in the cabinet, but is equipped with a small bright tin reflector at the back, in order to focus or concentrate the light. The patient is placed in a recumbent position on a cot and the lamp, which is suspended from the ceiling, is held near him. The light is then converged or directed against the affected part. This method is especially effective in cases of acute or chronic rheumatism in which the disease has so crippled the patient as to render it painful or difficult for him to get in and out of the cabinet. The arc lamp with reflector is used in such instances and always produces the most comforting sensation and satisfactory results. In pursuing the light treatment in such cases, the original cause of the disease is not lost sight of, and appropriate drug treatment is administered simultaneously.

I attribute my restoration to comfort and usefulness to the local application of light. It brought me, I may say, out of a living death, and it has been the means in my hands of securing similar results in other cases of locomotor ataxia. If the affection is not too far advanced, I am confident that benefit will be conferred in all cases. The chief difficulty lies in the inability to secure the cases at the time when the most good can be accomplished, drug treatment having been persisted in despite negative results and positive advance in the march of the disease. Who has seen a case of locomotor ataxia cured by mercury and iodide of potash? These agents do not restore cells that are lost. I believe that the light properly and timely applied will do so. I have seen it in my own and similar cases.

To the general practitioner the great length of time and the personal supervision of the treatment required are serious drawbacks which are overcome in the instance of the physician who gives his time and his best efforts to this special therapeutic procedure. I am convinced that we possess in it a mechanism of inestimable value, and the visible results in my own person attest the truth of this statement.

An account of Nils Finsen and his work can be found in *McClure's Magazine* for May, 1903.

---

### WHEN NOT TO OPERATE FOR APPENDICITIS.

J. E. Moore, Minneapolis (*Journal A. M. A.*, June 24), combats the dictum that all cases of appendicitis are operative cases, holds that while certain classes of cases, such as chronic appendicitis without acute attacks, those with localized abscesses, and acute cases seen in the beginning of the attack, call for operation provided hospital facilities and a good surgeon are available; there are others, he believes, in which surgical interference is not advisable. The conditions in which the radical operation is not the best treatment as summarized by him as follows: "First, when the patient is evidently moribund; second, when the patient is evidently convalescing; third, when certain grave complications are present; fourth, in the midway cases beginning with the third day when the physician and surgeon are in doubt; fifth, in the extreme cases of suppurating peritonitis." Even in serious complications, or when general anesthesis is contradicted by the condition of the patient, if localized abscess exists, he states that it should be evacuated under local anesthesia. Ochsner's starvation treatment, Moore says, has been badly misunderstood. Ochsner did not recommend starvation and lavage for appendicitis, but for spreading peritonitis due to neglected appendicitis. In such cases, Moore, by opening abscesses locally and by using to a greater or less extent Ochsner's method, has been able to tide them over to a successful interval operation.

